# Reduced expression of tissue factor pathway inhibitor-2 contributes to apoptosis and angiogenesis in cervical cancer

**DOI:** 10.1186/1756-9966-31-1

**Published:** 2012-01-02

**Authors:** Qiao Zhang, Yao Zhang, Shi Z Wang, Ning Wang, Wei G Jiang, Yao H Ji, Shu L Zhang

**Affiliations:** 1 Department of Obstetrics and Gynecology, Shengjing Hospital of China Medical University, Shenyang(110004), China; 2 Department of Pathology, Shengjing Hospital of China Medical University, Shenyang (110004), China; 3 Virus Laboratory, Shengjing Hospital of China Medical University, Shenyang (110004), China

**Keywords:** cervical cancer, Tissue factor pathway inhibitor-2, immunohistochemical study, apoptosis, angiogenesis

## Abstract

**Background:**

Tissue factor pathway inhibitor-2 (TFPI-2) is an extracellular matrix associated broad-spectrum Kunitz-type serine proteinase inhibitor. Recently, down regulation of TFPI-2 was suggested to be involved in tumor invasion and metastasis in some cancers.

**Methods:**

This study involved 12 normal cervical squamous epithelia, 48 cervical intraepithelial neoplasia (CIN), and 68 cervical cancer. The expression of TFPI-2, Ki-67 and vascular endothelial growth factor (VEGF) were investigated by immunohistochemistry staining. The apoptolic index(AI) was determined with an in situ end-labeling assay(TUNEL). And the marker of CD34 staining was used as an indicator of microvessel density (MVD).

**Results:**

TFPI-2 expression has a decreasing trend with the progression of cervical cancer and was significantly correlated with FIGO stage, lymph node metastasis and HPV infection. In addition, there were significant positive correlations between the grading of TFPI-2 expression and AI(P = 0.004). In contrast, the expression of TFPI-2 and VEGF or MVD was negatively correlated (both p < 0.001). However, we did not establish any signiﬁcant correlation between Ki-67 and TFPI-2 expression in cervical cancer.

**Conclusions:**

The results suggested that the expression of TFPI-2 had a decreasing trend with tumor progression of cervical cancer. There was a close association between the expression of TFPI-2 and tumor cell apoptosis and angiogenesis in patients with cervical cancer. TFPI-2 may play an inhibitive role during the development of cervical cancer.

## Background

Cervical cancer is the second most common malignancy in women around the world [1]. Cervical cancer occurs in a multi-step process, a sequential transition from a cervix with a normal epithelium to cervical intraepithelial neoplasia (CIN) and invasive cervical cancer. It is clear that persistent high-risk Human Papillomavirus (hr-HPV) infections are the strongest epidemiologic risk factor for the development of invasive cervical cancer [2]. However, HPV infection alone is not sufﬁcient to cause cervical cancer. Consequently, much interest has been focused on the molecular basis which contribute to drive the progression of cervical cancer. Proteolytic degradation of the extracellular matrix (ECM) is considered to be an essential step in tumor growth and metastasis. Tissue factor pathway inhibitor-2 (TFPI-2), a 32-kDa broad-spectrum Kunitz-type serine proteinase inhibitor, abundantly produced by a variety of human tissues and directionally secreted into their ECM [3-5]. TFPI-2 is thought to negatively regulate the enzymatic activity of ECM-associated trypsin, plasmin, and VIIa-tissue factor complexly to protect the ECM stability [6].

In humans, TFPI-2 gene is located on chromosome 7q22, and consists of three Kunitz-type serine proteinase inhibitory domains similar to the classical tissue factor pathway inhibitor (TFPI-1). While the first Kunitz-type domain of TFPI-2 appears to contain the main inhibitory activity towards a number of serine proteinases [7]. The degradation of ECM involves a variety of proteases, particularly metalloproteinases (MMPs). MMPs take part in virtually all events of ECM remodeling. It is reported that upregulation the expression of MMPs strongly associated with the progression of several malignancies, including cervical cancer [8]. TFPI-2 has also been reported to effectively regulate MMPs activity by inhibiting activation of proMMPs by trypsin-like serine proteinases [9]. TFPI-2 gene promote contains a complete CpG island region of at least 220-bp. It is observed that the TFPI-2 expression, decreasing or even diminishing, attributed to promoting hypermethylation in nasopharyngeal carcinoma [10]. Recent ﬁndings suggest decreasing TFPI-2 expression plays a significant role in inhibiting cell migration and tumor invasion by a mechanism that involves its inhibitory activity [11,12]. In addition, it is revealed that aberrant methylation of TFPI-2 was present in a high proportion of cervical cancer clinical samples and cell lines [13,14]. Thus, TFPI-2 might be a target gene in cervical cancer. However, the expression of TFPI-2 has not yet been examined in cervical tissues.

In this study, we investigated TFPI-2 expression in cervical lesions by immunohistochemical staining. We then studied the correlation between TFPI-2 and apoptosis, ki-67, VEGF and MVD expression to evaluate whether TFPI-2 contributed to tumor cell apoptosis, proliferation and angiogenesis in patients with cervical cancer.

## Materials and methods

### Specimens

A total of 128 uterine cervical samples was collected from patients who had undergone surgery at Shengjing Hospital (Shenyang City, Liaoning Province, PR.China) between 2009 and 2010. The specimens included 48 cervical intraepithelial neoplasia (CIN) and 68 invasive cervical cancer(ICC), along with 12 normal squamous epithelial specimens. The median age of all the patients was 43 years (range 22-71 years). The normal squamous epithelial specimens were collected from uteri of patients who had undergone hysterectomy without malignancy. Ths study was approved by the Ethics Committee of China Medical University University. Informed written consent was obtained from all subjects prior to the study.

The histopathological diagnosis was based on World Health Organization classifications, and the clinical staging was defined according to the International Federation of Gynecology and Obstetrics (FIGO) clinical staging system. All the subjects had complete clinical and pathological data, and none received preoperative radiotherapy, chemotherapy and biological therapy before surgery.

### Immunohistochemical staining(IHC)

The specific antibodies against TFPI-2 was purchased from Biosynthesis Biotechnology co. (Peking, China), Ki-67, VEGF, and CD34 were purchased from Zhongshan Goldenbridge Biotechnology co.(Peking, China).

Surgically resected tissue samples were routinely fixed in 10% formalin solution, paraffin-embedded, and cut into 4-μm-thick sections. After deparaffinization and rehydration, the sections were heated in three 5-minute periods in microwave oven at 100°C with sodium citrate buffer (10 mM; pH 6), cooled down in the same buffer at room temperature, and subsequently incubated 20 min with 3% hydrogen peroxide. The antibodies for TFPI-2, Ki-67, VEGF and CD34 were used at 1:200, 1:100, 1:100 and 1:100, respectively. The serial sections were incubated with primary antibodies in a humid chamber at 4°C overnight. Sections were washed three times in phosphate-buffer solution (PBS) and further incubated with a biotinylated secondary antibody for 30 min at room temperature. Streptavidin-horseradish peroxidase conjugate was added and the peroxidase activity was made visible with diaminobenzidine and counterstained with hematoxylin for 30 sec. As a control experiment, we performed an identical immunohistochemical procedure with omission of the primary antibody.

### TUNEL assay

Apoptosis of tumor sections was detected by TUNEL assay using the In Situ Cell Death Detection Kit, POD which was purchased from Roche (Mannheim, Germany). According to the manufacturer's instructions, after routine deparaffinisation, sections were digested with proteinase K working solution at room temperature for 15 minutes and washed twice with PBS. TUNEL reaction mixture was prepared. The sections were incubated with 50 μl TUNEL reaction mixture each for 60 min at 37°C in a humidified atmosphere in the dark. Sections were rinsed 3 times with PBS and further incubated with Converter-POD in a humidified chamber for 30 min at 37°C. After the sections were washed with PBS for 3 times, DAB was used as chromogen and sections were counterstained with Hematoxylin.

### HPV testing

The cervical swab samples were collected and transported using the PreservCytR LBC medium (Cytyc, Bedford, MA, USA). Samples may be held up at a temperature between 2°C and 8°C and shipped to the testing laboratory, a preservative has been added to the Transport Medium to retard bacterial growth and to retain the integrity of DNA. Test of type HPV was carried out by the Virus Laboratory, Shengjing Hospital (Shenyang City, Liaoning Province, PR.China) using the HPV GenoArray test kit (HybriBio, Hong Kong) according to the manufacturer's instructions. The GenoArray test is capable of amplifying 21 HPV genotypes: 13 HR types (16, 18, 31, 33, 35, 39, 45, 51, 52, 56, 58, 59, and 68), 5 LR genotypes (6, 11, 42, 43, and 44), and 3 types common in China (53, 66, and CP8304).

### Grading of immunostaining

Afterwards, the results of immunostaining were mounted and examined using a bright-field microscope by two independent observers without knowledge of the clinical data for each patient.

**Figure 1:**
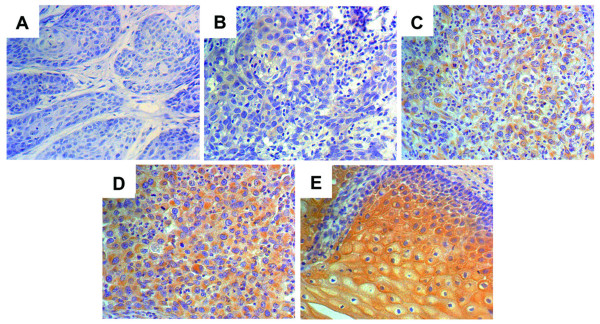
**The grading of immunohistochemical staining for TFPI-2**. Immunohistochemical staining of cervical tissues for TFPI-2 (A-E). The immunostaining intensity was deﬁned as grade 0 (no detectable staining, A), grade1 (weak staining, B), grade 2(clear but not so strong staining, C), grade 3 (more strong staining, D) and grade 4 (stronggest staining, E). The nuclei were counterstained with hematoxylin blue. Image magnifications are 200×.

For assessing the immunostaining, we used a semiquantitative approach to grade the TFPI-2 protein staining intensity as follows. The strongest staining was set at 100% and the staining intensity was rated as follows: 75% to 100% (++++), 50% to75% (+++), 10 to 50% (++), and < 10% (+) (Figure 1). The VEGF expression in the tumor cells was also evaluated using a semi-quantitative scoring system: 0 for absence of immunostaining(-), 1 for light staining(+), 2 for moderate staining(++), and 3 for heavy staining(+++). All TUNEL signal positive or Ki-67 immunolabelling nuclei were then counted from the total number of at least 2000 tumor cells in randomly selected fields in each case. In CIN lesions, these counting procedures were performed in the whole epithelial layers. The apoptotic cell index (AI) and Ki-67 labeling index (PI) were calculated as number per 100 cells. Microvessel density (MVD) was determined by counting the number of vessels plus immunoreactive endothelial cells per 200× high power field in the area of the most intense vascularization (hot spot) of each tumor, and the average count was recorded.

### Statistical analysis

Statistical analysis was performed using the SPSS 17.0 program package. Mean values were compared with unpaired Student's t-test or one-way ANOVA analysis, and categorical variables were compared with Fisher's Exact Test. The Chi-square linear trend test was used to check for correlation between TFPI-2 positive expression and clinicopathologic factors. The Spearman's correlation test was used to analyze consistency level between TFPI-2 and AI, PI, VEGF or MVD. The Kruskal-Wallis H test was used to analyze the association between the intensity of TFPI-2 immunoexpression and HPV infection. For the sake of statistical convenience, the positive results of -, +, ++, +++ and ++++ were scored as 0, 1, 2, 3 and 4. Two sided P-values less than 0.05 were considered statistically significant.

## Results

### Patient characteristics

Immunohistochemical analysis was performed on 128 pathological cervical neoplasms, including 48 CIN and 68 ICC, and along with 12 normal squamous epithelial specimens. Patient characteristics were presented in Table 1.

**Table 1:** Clinical and pathological characteristics


**Characteristics**	**Number of cases (%)**

Range	22-71(years)
Average	43 (years)
Samples	
normal squamous epithelial specimens	12 (9.4)
cervical intraepithelial neoplasms (CIN)	48(37.5)
CIN I	21 (43.7)
CIN II/III	27(56.3)
invasive CC(ICC)	68(53.1)
well-differentiated(WICC)	13(19.1)
moderately differentiated(MICC)	39(57.4)
poorly differentiated(PICC)	16(23.5)
Histology	
Squamous cell carcinoma(SCC)	61(89.7)
Adenosquamous cell carcinoma(ACC)	7(10.3)
Figo stage	
Ia	9(13.2)
Ib	28(41.2)
IIa	21(30.9)
IIb	10(14.7)
Lymph nodes metastasis(LN)	
Absent	51(75)
Present	17(25)
HPV infection	
Absent	38(29.7)
Present	90(70.3)

### Expression of TFPI-2 in cervical neoplasms

**Figure 2:**
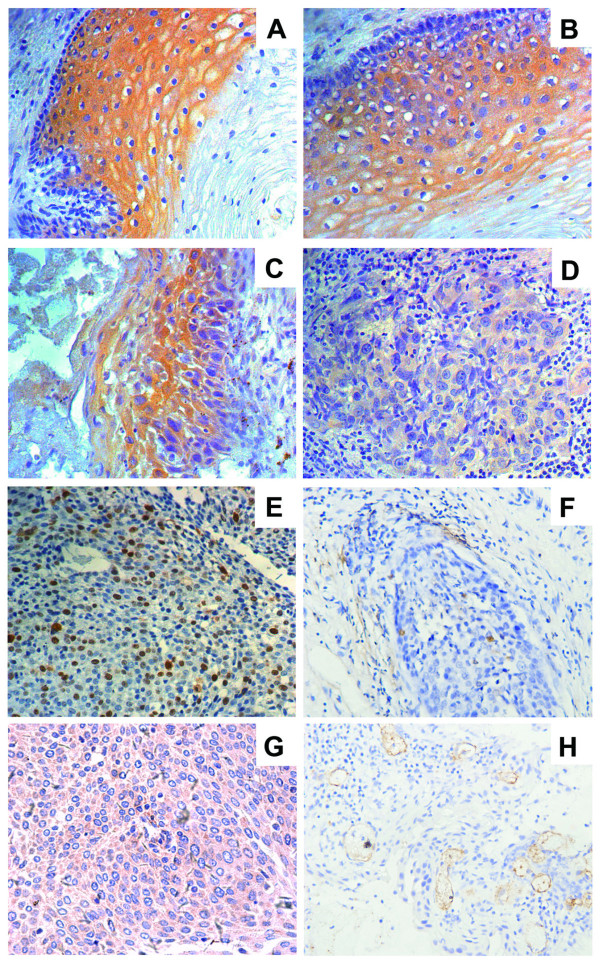
**Immunohistochemical staining of TFPI-2, and Ki-67, TUNEL, VEGF and CD34 in cervical tissues**. Immunohistochemical staining of TFPI-2 in cervical tissues (A-D), and Ki-67 (E), TUNEL (F), VEGF (G) and CD34 (H) in ICC. The analysis showed TFPI-2 expression in normal squamous epithelial cells showed strongly positive staining for cytoplasmic(A), clear cytoplasmic staining in CIN I (B), while CIN II and III show potent staining (C), weak staining in tumor cells (D). The nuclei were counterstained with hematoxylin blue. Image magnifications are 200×.

We observed TFPI-2 was expressed only in the cytoplasm of the cervical tissues. All normal squamous epithelial cells showed potent immunostaining for cytoplasmic TFPI-2 (Figure 2A), while the staining for cytoplasmic TFPI-2 was lower in ICC (Figure 2D). In CIN, the immunostaining of cytoplasmic TFPI-2 was clear but not so strongly observed. Cytoplasmic TFPI-2 immunostaining in CIN I was potent (Figure 2B), while that in CIN II and III was weak (Figure 2C). Clearly, the immunostaining of TFPI-2 decreased together with tumor progression, this being statistically significant (p < 0.005).

Cells undergoing apoptosis is a form of programmed cell death characterized leading to apoptotic bodies. TUNEL signals were detected not only in these cells but also in morphologically viable cells at the start of apoptosis, as identified by distinct nuclear staining(Figure 2F). Ki-67 staining was expressed in the nuclei of the cervical tissues(Figure 2E). Immunohistochemical staining of VEGF is mainly distributed in the cytoplasm of epithelial cells of the cervix(Figure 2G). The immunoreactivity of anti-CD34 antibody was located only on the cytoplasm of endothelial cells, and not on tumor cells or interstitial cells(Figure 2H).

### Correlation between clinicopathologic factors and TFPI-2 expression

Data on the correlation between clinicopathologic factors and the grading of TFPI-2 expression are summarized in Table 2. Grading of expression of TFPI-2 was significantly associated with histopathological, FIGO stage, lymph node metastasis and HPV infection.

**Table 2:** Correlation between clinicopathologic factors and TFPI-2 expression


**Characteristics**	**n**	**TFPI-2**					**P**

		**-**	**+**	**++**	**+++**	**++++**	

normal	12	0	0	0	2	10	< 0.001
CIN	48	0	3	19	18	8	
CIN I	21	0	0	3	10	8	< 0.001
CIN II/III	27	0	3	16	8	0	
ICC	68	23	25	19	1	0	
WICC	13	2	5	6	0	0	0.474
MICC	39	13	15	10	1	0	
PICC	16	8	5	3	0	0	
Histology							
SCC	61	19	22	19	1	0	0.304
ACC	7	4	3	0	0	0	
Figo stage							
I	37	5	17	13	1	0	0.003
II	31	18	8	6	0	0	
LN metastasis							
Absent	51	13	19	18	1	0	0.037
Present	17	10	6	1	0	0	
HPV status							
Absent	38	1	6	9	9	13	< 0.001
Present	90	26	22	25	12	5	

The proportion of grading expression of TFPI-2 have a decreasing trend from normal, CIN to ICC, indicating that the expression of TFPI-2 have an association and linear relationship with the increase of malignant potential of cervical neoplasia. The expression of TFPI-2 in CIN II and III was significantly lower than that in CIN I, indicating that the decreased TFPI-2 expression may link to the increase of malignant potential of CIN. But the decreasing trend of grading proportion was not observed.

Further, we analyzed there was no significant difference between the expression of TFPI-2 and differentiation or histology. In contrast, the grading expression of TFPI-2 was significantly having a decreasing trend with FIGO stage, lymph node metastasis and HPV infection.

Thus, further examinations were done to analyze more precisely the level of TFPI-2 in HPV infection by using Kruskal-Wallis H Test. The proportion of TFPI-2 expression variations between HPV infected and non-infected cases revealed that TFPI-2 expression in the HPV positive samples was significantly lower compared to HPV negative samples. Further, we divided the patients with HPV infected into four groups, as Normal, CIN I, CIN II/III and ICC. The relationship between TFPI-2 expression and these HPV positive samples in these four groups was significant (p < 0.001).(Table 3)

**Table 3:** Association between HPV infection and TFPI-2 expression in normal and neoplastic cervical epithelium

	n	HPV-positive	TFPI-2				
			**-**	**+**	**++**	**+++**	**++++**

Normal	12	3	0	0	2	2	1
CIN I	21	11	0	0	1	6	4
CIN II/III	27	18	0	2	12	4	0
ICC	68	58	22	20	16	0	0

### Correlation between TFPI-2 and apoptosis, ki-67, VEGF and MVD expression

The analysis was done to clarify whether there is difference of AI, PI, VEGF and MVD according to TFPI-2 positive and negative samples. As shown in Table 4, TFPI-2 negative AI in ICC is lower than the expression of TFPI-2 positive ICC. The VEGF and MVD in the TFPI-2 positive samples was significantly lower compared to TFPI-2 negative samples in ICC. However, there was no signiﬁcant correlation of PI between TFPI-2 positive and negative samples.

**Table 4:** Correlation between TFPI-2 status and and AI, PI, VEGF and MVD during malignant grading


	**AI**		**PI**		**VEGF**		**MVD(mean ± SD)**	

	**TFPI-2** **(+)**	**TFPI-2** **(-)**	**TFPI-2** **(+)**	**TFPI-2** **(-)**	**TFPI-2** **(+)**	**TFPI-2** **(-)**	**TFPI-2** **(+)**	**TFPI-2** **(-)**

Normal	0a	-	11.3a	-	0.25a	-	30.5 ± 12.5a	-
CIN I	0.12a, b	-	20.1a, b	-	0.38a, b	-	36.1 ± 7.9a, b	-
CIN II/III	1.13a, c	-	50.8c, d	-	0.59a, b	-	42.6 ± 24.3a, b	-
ICC	2.41	1.8	57.5	64.7	1.2	2.2	63.5 ± 19.3	69.8 ± 21.0
P*		0.001		0.054		< 0.001		0.033

ap < 0.001 when compared to ICC; bp > 0.05 when compared to normal cervix;and cP < 0.001 when CIN I compared to CIN II/III; dP = 0.005 when CIN II/III compared to ICC; P* when TFPI-2-negative compared to TFPI-2-positive. The TFPI-2 positive results of +,++,+++ and ++++ were merged into one group.

Thus, new experiments were done to analyze more precisely the level of AI, LI, VEGF and MVD in normal epithelial specimens, CIN, and ICC of TFPI-2 positive samples. The AI clearly increased together with tumor progression in the TFPI-2 positive samples, this being statistically significant. The PI in CIN II and III and ICC were signiﬁcantly higher than those in normal epithelium. There was however no signiﬁcant difference between CIN I and normal epithelium. The VEGF in ICC were also signiﬁcantly higher than CIN and normal epithelia, and there was no difference between CIN and normal epithelium. The MVD was similar to VEGF.

Then, in order to analyze the consistency level between the grading of TFPI-2 expression and AI, PI, VEGF or MVD, 68 ICC samples were classified as -, +, ++ and +++ four groups. Spearman rank correlation test was used. The data on the correlation are summarized in Table 5. As a result, there were significant positive correlations between the grading of TFPI-2 expression and AI. In contrast, the expression of TFPI-2 and VEGF or MVD was negatively correlated. But to PI, this trend of statistical significance was not observed.

**Table 5:** Correlation between the grading expression of TFPI-2 and AI, PI, VEGF and MVD in ICC

TFPI-2	n	AI	PI	VEGF	MVD(mean ± SD)
-	23	1.8	64.7	2.2	69.8 ± 21.0
+	25	2.2	58.9	1.5	64.8 ± 19.2
++	19	2.5	56.6	0.8	62.3 ± 18.2
+++	1	4.8	39	0	54.4 ± 9.4
R		0.346	-0.202	-0.552	-0.767
P		0.004	0.098	< 0.001	< 0.001

## Discussion

Human TFPI-2, also known as placental protein (PP5) and matrix-associated serine protease inhibitor (MSPI), is an ECM-associated Kunitz-type serine proteinase inhibitor [15]. TFPI-2 plays an important role in normal ECM remodeling, and is also becoming increasingly recognized as a tumor suppressor gene. In several types of malignancies, such as choriocarcinoma [16], glioma [17], prostate cancer [18], pancreatic carcinoma [19] and lung cancer [20], TFPI-2 has significantly demonstrated tumor-suppressive functions during tumor cell invasion, metastasis, apoptosis, proliferation and angiogenesis. It was reported that, TFPI-2 showed high frequency of CpG islands aberrantly methylated in both cervical cancer specimens and cell lines [13,14]. But, to our knowledge, little is known on the role of TFPI-2 silencing in cervical cancer. To investigate the relationship between TFPI-2 and tumor cell apoptosis, proliferation and angiogenesis in patients with cervical cancer, we analyzed the immunohistochemical expression levels of TFPI-2, with relationship to AI, PI, VEGF and MVD in cervical biopsy tissues. Our data suggested that TFPI-2 inhibited tumor apoptosis and metastasis of cervical cancer and might be a regulatory molecule in the malignant potential of cervical cancer.

In the present study, we found that TFPI-2 expression in all patients with normal epithelial cells and CIN was positive, while that was activated in 66.2% of cervical carcinomas in immunohistochemical analysis. Our data demonstrated that the grading expression of TFPI-2 had a decreasing trend with the increase of malignant potential of cervical neoplasia. Similarly, immunoexpression of TFPI-2 has been studied in many other different tumors (laryngeal, breast, gastric, colon, pancreatic, renal, endometrial cancer and glial neoplasms) and the expression of TFPI-2 diminished with an increasing degree of malignancy [21]. Wong et al analyzed the mRNA expression of TFPI-2, their data suggested that when compared with the corresponding nontumorous livers, TFPI-2 was significantly under-expressed in approximately 90% of primary hepatocellular carcinomas [11]. It has also been reported that there was a good correlation between the immunoexpression of TFPI-2 staining score and mRNA levels measured by real-time PCR [11,22]. Thus, the expression of TFPI-2 was decreased in some tumors, which was consistent with the results of our study. The mechanism of downregulation of TFPI-2 expression during tumor progression was signiﬁcantly correlated with the promoter aberrant methylation. It is demonstrated that the downregulation of TFPI-2 expression was signiﬁcantly correlated with the promoter hypermethylation in some cancer lesions and cell lines, such as nasopharyngeal carcinoma [10], hepatocellular carcinoma [11], lung cancer [22] and breast cancer [23].

We further analyzed the correlation of TFPI-2 expression and clinicopathologic factors of patients, to investigate whether the expression of TFPI-2 could predict increased risk of metastasis and malignancy. Our data indicated that the grading of TFPI-2 gene expression had a decreasing trend with FIGO stages, lymph node metastasis and HPV infection of cervical cancer. Our results were similar to the study of non-small-cell lung cancer, in which the downregulation of TFPI-2 mRNA was more frequently associated with advanced stages. It was observed in stage I-II NSCLC (11/33, 33%) and stage III-IV(11/26, 42%)[22].

There is no doubt that HPV infection is the most important risk factor for the development of cervical cancer [24]. But progression of an HPV-infected cervical intraepithelial neoplastic to invasive cervical cancer is infrequent. There are some other factors that influence the susceptibility of HPV infection and drive progression of HPV-induced neoplastic to invasive cervical cancer [25]. Alessandro et al reported that the expression of TFPI-2 downregulation in HPV16 and HPV18-infected stage IB-IIA cervical cancers compared to normal cervical keratinocyte cultures [14]. We also observed that the grading of TFPI-2 expression in the HPV positive samples was significantly lower compared to HPV negative samples. Thus, TFPI-2 expression in cervical lesions maybe correlates with the HPV activity.

These results suggest that the transcriptional repression of human TFPI-2 may have an important role during the genesis or progression of cervical carcinoma. It becomes of importance to clarify the role of TFPI-2 expression in cervix epithelial cells.

In the current study, we found that the AI clearly increased together with tumor progression. In fact, loss of AI has been suggested to be involved in malignant transformation [26]. In addition, the data showed that apoptosis was associated with TFPI-2 in cervical carcinoma. The expression of TFPI-2- negative AI was lower than TFPI-2 positive. We also found that there were significant positive correlations between the grading of TFPI-2 expression and AI by Spearman's correlation test. These data suggested that the diminish expression of TFPI-2 in cervical cancer is associated with a decrease in apoptosis. George et al reported that restoration of TFPI-2 in U-251, a highly invasive human glioblastoma cell line, activated both intrinsic and extrinsic caspase-mediated, pro-apoptotic signaling pathways and induced apoptosis in these cells [27]. Further, Prakasha et al reported that both TFPI-2 and R24K KD1, whose mutated ﬁrst Kunitz-type domain, activated the signaling pathways resulting in apoptosis, and their data suggested that TFPI-2's serine proteinase inhibitory activity may play a role in this process [28]. Thus, the findings suggested that TFPI-2 play an important role with apoptosis in cervical carcinoma.

It is clear that VEGF dominantly expresses via a paracrine pathway to surrounding microvessels in tumor cells, and VEGF expression is critical for microvessel density in malignancy [29]. In the current study, the expression of TFPI-2 and VEGF was negatively correlated. Therefore, we believe that decreased TFPI-2 expression correlates with increased expression of VEGF in cervical carcinoma, suggesting that active TFPI-2 plays a suppressive role on VEGF gene expression. Hitendra et al stably transfected HT-1080 ﬁbrosarcoma cells expressing active human TFPI-2, revealed that TFPI-2 could regulate tumor angiogenesis by reducing synthesis of the VEGF receptor [30]. There is growing evidence suggesting that TFPI-2 is critically involved in the progression of angiogenesis [12,31]. We also found that the VEGF expression and MVD in the TFPI-2 positive samples was significantly lower compared to TFPI-2 negative samples. Such result indicated that Human TFPI-2 may inhibit VEGF-stimulated capacity of angiogenesis in the development of cervical cancer, which leads to unlimited the growth of tumors.

The Ki67 antigen is a nuclear nonhistone protein to be expressed throughout the cell cycle, except G0. In the present study, we used Ki-67 immunohistochemistry to determine the cell proliferative activity. We observed that there was no signiﬁcant correlation between PI and TFPI-2 expression in invasive cervical cancer. Our findings contrast with previous studies in vitro, which demonstrated that ectopic expression of TFPI-2 significantly inhibited cell proliferation in hepatocellular carcinoma [11], nasopharyngeal carcinoma [10] cell lines and Human retinal endothelial cells [32]. These differences may be due to variation in cell type-specific responses, or the detection of an extensive cell cycle phase by Ki-67 immunohistochemistry, and/or our ability to examine complex in lesions. And further study will be essential for discovering more valuable information about TFPI-2 expression and cell proliferation in cervical carcinoma.

## Conclusions

In conclusion, our data shows the expression of TFPI-2 in cervical lesions has a decreasing trend with tumor progression. It is believed that TFPI-2 contributes to tumor cell apoptosis and angiogenesis in patients with cervical cancer. TFPI-2 may be considered as a tumor suppressor gene during the development of cervical cancer. As a result, we propose that TFPI-2 silencing was probably one of the mechanisms of cervical cancer. Future studies focused on the molecular mechanism whereby TFPI-2 expression and function affects tumor cell gene expression of cervical cancer are needed.

## Competing interests

The authors declare that they have no competing interests.

## Authors' contributions

QZ and SZ designed the study and drafted the manuscript; QZ and YZ carried out the Immunochemistry assay; SW participated in the TUNEL assay; NW participated in data organization and statistical analysis; WJ collected the cervical biopsy samples and accomplished pathological diagnosis; YJ carried out the HPV testing. All authors read and approved the final manuscript.
